# EBV-Positive Nodal T- and NK-Cell Lymphoma Mimicking Anaplastic Large Cell Lymphoma: A Case Report

**DOI:** 10.3390/hematolrep16020031

**Published:** 2024-05-23

**Authors:** Brooj Abro, Pamela Allen, Saja Asakrah, Kyle Bradley, Linsheng Zhang

**Affiliations:** 1 Department of Pathology and Laboratory Medicine, Emory University School of Medicine, 1364 Clifton Road NE, Atlanta, GA 30322, USA; 2 Department of Hematology and Oncology, Winship Cancer Institute of Emory University, 1365 Clifton Road NE, Atlanta, GA 30322, USA

**Keywords:** Epstein–Barr Virus (EBV), lymphoma, EBV-positive nodal T- and NK-cell lymphoma, anaplastic large cell lymphoma

## Abstract

EBV-positive nodal T- and NK-cell lymphoma (EBV+ NT/NKCL) is a recently recognized entity in the 5th edition of the WHO Classification of Tumors of Hematopoietic and Lymphoid Tissues. Notably, CD30 positivity is frequently observed in (EBV+ NT/NKCL), creating diagnostic challenges to distinguish it from ALK-negative anaplastic large cell lymphoma (ALCL). Furthermore, cases of EBV+ ALCL have been documented in the literature, predating the inclusion of EBV+ nodal cytotoxic T-cell lymphoma as a variant of peripheral T-cell lymphoma. We present a case of a 47-year-old male presenting with multiple lymphadenopathies. The histomorphologic and immunophenotypic features of the lymph node closely resemble ALK-negative ALCL, characterized by uniform CD30 expression and a subcapsular distribution of lymphoma cells. However, the lymphoma cells exhibit diffuse positivity for EBV, consistent with EBV+ NT/NKCL. A case of ALK-negative ALCL with an immunophenotype identical to the EBV-positive case is included for comparison. Given that EBV+ NT/NKCL represents an aggressive neoplasm requiring unique clinical management compared to ALK-negative ALCL, it is critical to accurately differentiate EBV+ NT/NKCL from ALK-negative ALCL with a cytotoxic T-cell immunophenotype.

## 1. Introduction

Epstein–Barr Virus (EBV)-positive nodal T- and NK-cell lymphoma (EBV+ NT/NKCL) is recognized as a distinct entity in the 5th edition of the World Health Organization (WHO) Classification of Tumors of Hematopoietic and Lymphoid Tissues (WHO-HAEM5) [1] and a provisional entity in the 2022 International Consensus Classification (ICC) [2]. EBV+ NT/NKCL primarily affects East Asians, particularly middle-aged to elderly individuals, with a higher incidence in males. Patients typically present with lymphadenopathy and advanced-stage disease with or without involvement of bone marrow or other extranodal sites. An association with immunosuppression may or may not be present. Its prognosis is generally poor and worse than that of peripheral T-cell lymphoma, not otherwise specified (PTCL-NOS) [3,4,5]. EBV+ NT/NKCL is distinct from extranodal NK/T-cell lymphoma (ENKTL) because of its unique immunophenotypic features and genetic profile [1,2]. Given that CD30 positivity is observed in a significant proportion of EBV+ NT/NKCL cases [6], it can be challenging to distinguish it from ALK-negative anaplastic large cell lymphoma (ALCL). Cases of EBV+ mature T-cell lymphoma diagnosed as systemic ALK-negative ALCL have been reported in the literature [7], further complicating the differential diagnosis.

Systemic ALK-negative ALCL is a heterogeneous entity that is primarily diagnosed based on morphologic and immunophenotypic features. The characteristic morphologic features include a cohesive sinusoidal growth pattern of tumor cells and anaplastic cytomorphology with so-called hallmark cells that display horseshoe-shaped or leaf-like large nuclei. The defining immunophenotypic feature is strongly and uniformly positive CD30 with expression of some, but not all, mature T-cell markers [8]. A few characteristic genetic alterations have been identified in a subset of ALK-negative ALCL cases, which can aid in diagnosis. These include chromosomal rearrangement involving *DUSP22* in 20–30% cases, associated with overall favorable prognosis, and rearrangement of *TP63* in 5–8% cases, associated with poor clinical outcomes [9]. Recently, cases with strong diffuse CD30 expression and anaplastic morphology resembling Hodgkin-like cells with expression of CD15 have been documented in PTCL [10]. These cases characteristically carry a *JAK2* rearrangement. In cases lacking characteristic gene rearrangements, it can be challenging to differentiate them from CD30-expressing PTCL-NOS. The WHO-HAEM5 recommends classifying these cases conservatively as PTCL-NOS when there are doubts [8]. Notably, a cohesive sinusoidal infiltrate; uniformly and strongly positive staining for CD30; expression of CD56, EMA, clusterin, and cytotoxic markers, including TIA1, granzyme B, and perforin; and loss of CD43 and CD45 expression favor the diagnosis of ALCL.

As our understanding of the pathobiology of EBV+ mature T-cell lymphoma and ALK-negative ALCL advances, the classification of these two entities has been better defined [9]. In this report, we present a case of EBV+ NT/NKCL exhibiting morphologic and immunophenotypic features resembling ALK-negative ALCL. In addition, we include a case of ALK-negative ALCL with an identical immunophenotype for comparison. An approach to the differential diagnosis will be discussed, with a brief review of previously reported cases of EBV+ ALCL. By exploring the pathologic characteristics and diagnostic challenges associated with EBV+ NT/NKCL and ALK-negative ALCL, this case report aims to contribute to the current understanding of these lymphomas and facilitate an accurate diagnosis to avoid diagnostic pitfalls and inappropriate clinical management.

## 2. Case Presentation

### 2.1. Case 1 (EBV+ NT/NKCL)

A 47-year-old male patient presented with fever and skin color changes in the digits of the left hand that persisted for 2 months. He later developed leg swelling and difficulty walking. He was hospitalized, and computerized tomography (CT) demonstrated lymphadenopathy in multiple regions, including axillary, subpectoral, supraclavicular, and jugular digastric chain lymph nodes, with the largest measuring 1.1 × 2.1 cm, as well as multiple bilateral, predominantly subpleural pulmonary nodules, with the largest measuring 0.7 × 1.1 cm. A complete blood count revealed the following: white blood cell count 20.9 × 10^−3^/microliter (reference range: 4.2–9.1), red blood cell count 3.51 × 10^−6^/microliter (reference range: 4.63–6.08), hematocrit 33.3% (reference range: 37.7–46.5%), and platelet count 549 × 10^−3^/microliter (reference range: 150–400).

**Figure 1:**
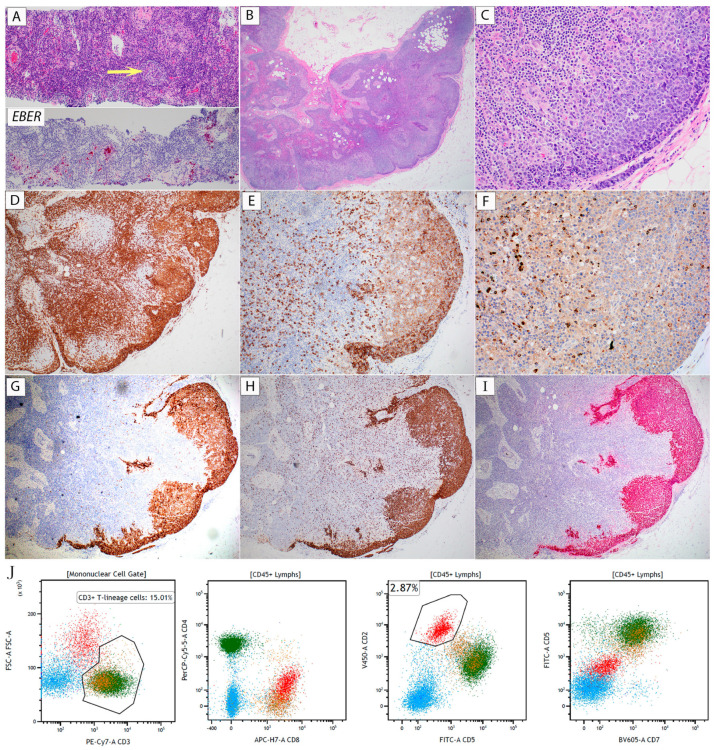
(Case 1, EBV+ NT/NKCL). (**A**) Core biopsy. Upper half: H&E (100×) shows relatively preserved architecture with a small benign-appearing lymphoid follicle (arrow). No obvious atypical lymphoid cells can be identified. Lower half: *EBER* in situ hybridization (100×) reveals a few small groups of positive cells. (**B**–**J**) Excision. (**B**) H&E (20×) shows mostly preserved lymph node architecture with a focal subcapsular infiltrate of atypical large cells. (**C**) H&E (200×) shows a monotonous population of large atypical cells, with some displaying prominent nucleoli. (**D**–**H**) Immunohistochemical stains (20× except (F)) were positive for (**D**) CD3, (**E**) CD8, (**F**) TIA-1 (40×), (**G**) CD30, (**H**) Ki-67, and (**I**) *EBER*. (**J**) Representative flow cytometry dot plots. The red-colored gate shows an abnormal cell population with high FSC, bright CD2, dim surface CD3, and dim to negative CD5 and CD7. Green: CD3+/CD4+ T-cells; orange: CD3+/CD8+ T-cells; Dodger blue: B-cells. The material and methods for diagnostic workup are detailed in the Supplemental Materials.

A left axillary lymph node core biopsy was performed and revealed lymphoid tissue with no significant architectural distortion on light microscopy (Figure 1A, upper half). Although not obvious by morphologic examination, immunohistochemical staining revealed a small focus of atypical large cells showing dim CD3 expression and lacking CD5 expression. EBV staining by in situ hybridization of Epstein–Barr virus-encoded small RNAs (*EBER*) highlighted small aggregates of EBV-positive large cells (Figure 1A, lower half). The focal area of atypical cells was lost in subsequent tissue sections, precluding further characterization. Flow cytometry identified no monotypic B-cell population; the T-cell population could not be evaluated due to low sample cellularity. Excision of the left axillary lymph node was subsequently performed. Large atypical cells were focally present in the subcapsular area on the hematoxylin and eosin (H&E)-stained sections (Figure 1B,C). Immunohistochemical staining (Figure 1D–G) showed that these cells were positive for CD2, CD3, CD8, CD30, TCRβ, GATA-3, partial CD15 (small subset), and TIA-1 (few cells), and had a high Ki-67 proliferation rate approaching 100% (Figure 1H). Large atypical cells were diffusely and strongly positive for *EBER* (Figure 1I). These cells were negative for CD4, CD5, CD7, CD56, TCRγ, PD1, and ALK1. Flow cytometric immunophenotyping revealed a distinct cell population with high forward light scatter that expressed CD2 (bright), CD3 (dim), CD8, CD15, CD45, and dim to negative CD5 and CD7. This population displayed no expression of CD4, CD26, or CD56 (representative dot plots are shown in Figure 1J). No neoplastic lymphocytes were detected in the peripheral blood by morphologic examination of a blood smear or by flow cytometry. EBV DNA, detected by quantitative polymerase chain reaction (PCR), was 91,500 IU/mL (reference range: 0–299).

A diagnosis of partial lymph node involvement by EBV-positive peripheral T-cell lymphoma was made. Further work-up revealed that atypical cells were also present in the cerebrospinal fluid (CSF) sample, with an immunophenotype similar to the lymphoma cells identified in the axillary lymph node by flow cytometry. A staging bone marrow biopsy revealed an overall unremarkable bone marrow morphology with normal trilineage hematopoiesis, no lymphoid aggregates, and no granulomas. Although a few scattered *EBER*-positive cells were identified in the bone marrow core biopsy, immunohistochemical staining for CD3 showed no large CD3-positive T cells or abnormal clusters of T cells. Furthermore, abnormal CD8-positive cells similar to those seen in the lymph node were not detected in the bone marrow by flow cytometry. Chromosome analysis revealed a normal male karyotype, 46,XY[20]. T-cell receptor gamma chain gene (TRG) rearrangement by polymerase chain reaction (PCR) detected a polyclonal pattern with no evidence of a monoclonal rearrangement.

The patient received chemotherapy with BV-CHEP (brentuximab vedotin, cyclophosphamide, doxorubicin (hydroxydaunorubicin), etoposide, and prednisone; see https://clinicaltrials.gov/ct2/show/NCT03264131 (accessed on 24 April 2024)), followed by CHOEP (cyclophosphamide, doxorubicin, vincristine, etoposide, and prednisone) and weekly rituximab. A post-treatment positron emission tomography–computed tomography (PET-CT) scan revealed no evidence of residual nodal or extranodal lymphomatous disease. However, post-treatment brain magnetic resonance imaging (MRI) revealed multifocal lesions in the brainstem and elsewhere, consistent with lymphoma involvement, although CSF cytology and flow cytometric analysis reported no neoplastic cells, and a bone marrow biopsy showed no evidence of EBV+ cells at the time. The patient was started on DHAP (dexamethasone, high-dose cytarabine, and cisplatin), alternating with high-dose methotrexate and intrathecal methotrexate with each DHAP cycle. Post-treatment brain MRI revealed punctate foci of residual enhancement in the dorsal medulla, no residual enhancement in other areas, and no new enhancing lesions. PET-CT confirmed a marked reduction in brain lesions (no longer evident). Many of the previously observed FDG-avid bilateral inguinal and left iliac chain lymph nodes were also resolved. However, there was a persistent, intense, FDG-avid, and enlarged left external iliac lymph node. The patient continued to receive high-dose methotrexate. Post-treatment PET-CT revealed significant progression of the disease, as evidenced by increasingly FDG-avid right thyroid nodules, numerous new and enlarging FDG-avid pulmonary nodules, hepatic lesions, and multistation lymphadenopathy. Although the EBV quantitation in blood samples decreased to <5000 IU/mL during chemotherapy, the level subsequently increased to 62,800 IU/mL, consistent with disease relapse. Chemotherapy with the P-Gemox regimen (Pegaspargase, Gemcitabine, oxaliplatin) [11] was initiated for the relapsed lymphoma. However, the patient showed a poor response and eventually succumbed to disease progression 17 months after the initial diagnosis.

### 2.2. Case 2 (ALK-Negative ALCL)

**Figure 2:**
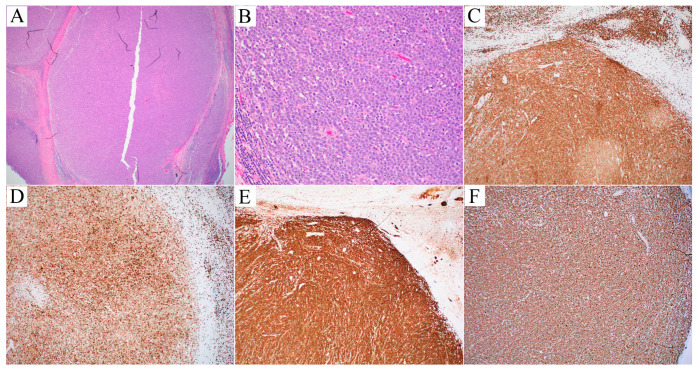
(Case 2, ALK-negative ALCL). (**A**,**B**) H&E stain. Low power ((**A**), 20×) shows a nodular proliferation of atypical lymphoid cells. At higher power ((**B**), 200×), the cytomorphologic features of the neoplastic cells are very similar to those seen in Case 1 (see Figure 1C). (**C**–**F**) (40×). Immunohistochemical stains showed the lymphoma cells to be positive for (**C**) CD3, (**D**) CD8, (**E**) CD30, and (**F**) Ki-67. The material and methods for diagnostic workup are detailed in the Supplemental Materials.

A 56-year-old male presented with persistent parotid and groin lymphadenopathy, fatigue, and night sweats, but denied fever, chills, or weight loss. CT imaging revealed an enlarged left inguinal lymph node measuring 3.2 × 3.1 cm, along with small lymph nodes in the suprainguinal region. PET-CT identified metabolic activity in the left neck with an SUV of 5.9, corresponding to an intraparotid lymph node measuring 1.9 × 1.9 cm. An inguinal lymph node excision was performed. H&E-stained sections of the lymph node showed near-complete effacement of the architecture by a nodular proliferation of large, pleomorphic lymphoid cells with prominent nucleoli and high mitotic activity (Figure 2A,B). Immunohistochemistry demonstrated positive staining for CD2, CD3 (Figure 2C), CD7 (weak, subset), CD8 (dim, Figure 2D), and CD30 (strong and diffuse, Figure 2E), while it was negative for CD4, CD5, CD20, CD25, CD56, ALK, and perforin. The Ki-67 proliferation index was high (90–100%, Figure 2F). An in situ hybridization of Epstein–Barr virus (*EBER*) was negative. Flow cytometric analysis confirmed the presence of an aberrant T-cell population expressing CD45, CD3 (decreased intensity), CD2 (increased intensity), and TCRαβ. Fluorescent in situ hybridization (FISH) identified no *ALK* or *P63* rearrangements and was inconclusive for *DUSP22* rearrangement. The patient was diagnosed with ALK-negative ALCL and received brentuximab vedotin in combination with CHP chemotherapy, with symptomatic improvement and resolution of the parotid lymph node. He subsequently received autologous hematopoietic stem cell transplantation and remained in complete remission 5 months after transplantation.

## 3. Discussion

Here, we present two cases of mature T-cell lymphoma with almost identical cytomorphologic and immunophenotypic features, with the exception of *EBER* positivity in one and not the other. Despite the subcapsular and sinusoidal distribution pattern and uniform expression of CD30, features characteristically associated with ALK-negative ALCL, the presence of diffusely positive *EBER* in the lymphoma cells in Case 1 indicates that this case should be classified as an EBV+ NT/NKCL with a T-cell phenotype. Classic Hodgkin lymphoma was ruled out based on the diffuse expression of the T-cell markers CD2, CD3, and CD8, as well as a complete lack of PAX5 expression. In contrast, although the lymphoma cells in Case 2 displayed the same immunophenotype as Case 1, in the absence of EBV, it was classified as ALK-negative ALCL.

EBV staining is not routinely included in the workup of ALCL, especially in patients without evidence of immunosuppression or immunocompromise. Consequently, EBV+ NT/NKCL with diffuse CD30 expression may pose a diagnostic pitfall. Typically, the neoplastic cells of EBV+ NT/NKCL are positive for at least one T-cell antigen (e.g., CD2, CD3, CD4, CD5, CD7, and CD8) and express cytotoxic molecules such as granzyme B, granzyme M, perforin, and/or TIA-1, but are negative for CD56, distinguishing them from ENKTL [3,12]. Before the inclusion of nodal EBV+ mature T-cell lymphoma as a variant of PTCL-NOS in the revised 4th edition WHO classification (WHO4RE) published in 2017, the classification of EBV+ mature T-cell lymphoma with strong uniform CD30 expression in tumor cells had been a topic of controversy, in part because the 4th edition WHO classification published in 2008 stated that in ALCL, ALK-negative is consistently negative for EBV. In a 2010 case report by Ma et al., 64 previously reported cases of non-cutaneous EBV+ ALCL were reviewed [7]. These cases were diagnosed based on uniform expression of CD30 and were predominantly found in immunocompetent patients. To our knowledge, no new cases of EBV+ systemic ALCL have been reported in the literature since the 2010 Ma et al. study. A case of cutaneous ALK-negative ALCL with diffuse EBV staining was reported by Gru et al. in 2019 [13]. The reported case was immune suppression-associated, expressed CD4 but not CD8 or TIA-1, and harbored a *BRAF* V600E mutation. Case 1 presented in this report would have been diagnosed as ALK-negative ALCL if an *EBER* stain had not been performed, underscoring the importance of including EBV staining, as well as a blood EBV titer test, in the diagnostic workup for cases suspected to be ALCL that exhibit a cytotoxic phenotype with CD8 expression and uniform CD30 expression. Furthermore, the current WHO HAEM5 lists EBV negativity as an essential criterion to establish the diagnosis of ALK-negative ALCL, indicating that EBV evaluation is now required to diagnose ALK-negative ALCL.

CD30 expression has been reported in over a third of adult T-cell leukemia/lymphoma (ATLL) cases, more frequently seen in the lymphomatous subtype (47%) [14] and associated with loss of FOXP3 expression [15]. ATLL cases usually express CD4 and CD25, which should trigger testing for human T-lymphotropic virus type 1 (HTLV-1). Occasionally, extracavitary primary effusion lymphoma (PEL, solid variant), which is frequently EBV+, expresses CD3 and CD30 in most neoplastic cells, mimicking ALCL. These cases have a plasmablastic immunophenotype with the expression of CD38, CD138, MUM1, and immunoglobulin light chain restriction, and PEL is defined by the expression of HHV8 [16]. Rarely, positive EBV staining in lymphoma cells can occur in ALCL or other mature T-cell lymphomas with secondary EBV infection. In these instances, unlike EBV+ NT/NKCL, EBV infection also involves non-neoplastic cells, and EBV positivity is also observed outside of the lymphoma components.

Gene expression profiling studies on EBV+ NT/NKCL have revealed upregulation of PD-L1 and T-cell related genes, along with downregulation of CD56, which is significantly different from ENKTL [17]. In a study by Wai et al., copy number changes, homologous recombination deficiency, and mutation profiles were compared among PTCL, ENKTL, and EBV+ NT/NKCL. The findings showed that EBV+ NT/NKCL exhibited significantly lower genomic instability and homologous recombination deficiency scores, and many immune-related pathways were significantly upregulated [18]. Other upregulated genes included NFκB-associated genes, BIRC3, NFKB1 (P50), and CD27. Additionally, studies on cytotoxic peripheral T-cell lymphoma, not specifically focused on EBV+ cases, revealed frequent mutations in epigenetic modifiers, such as *TET2*, *DNMT3A*, and genes involved in the JAK/STAT pathway [19]. Although these studies provided evidence to separate EBV+ NT/NKCL from ENKTL and PTCL-NOS, the JAK/STAT signal pathway mutation profile overlaps with that of ALK-negative ALCL [20]. Activation of the JAK/STAT pathway can be seen in two-thirds of ALK-negative ALCL cases, resulting from either gene mutations or gene rearrangements. A study from the European T-Cell Lymphoma Study Group identified a set of three genes (*TNFRSF8, BATF3*, and *TMOD1*) whose co-expression could potentially distinguish ALK-negative ALCL from PTCL-NOS, with an overall accuracy of approximately 97% in unrelated groups of patients [21]. Further investigation is required in order to determine the distinct genetic profile that can differentiate EBV+ NT/NKCL from ALK-negative ALCL. For our Case 1, given the diffuse EBV positivity observed in lymphoma cells, we did not pursue further molecular genetic studies for the diagnostic possibility of ALK-negative ALCL.

Although most patients with systemic ALK-negative ALCL present with stage III and IV disease and associated B symptoms, the prognosis is better than that of PTCL-NOS using conventional chemotherapy [22]. Anthracycline-containing multi-agent protocols have demonstrated high response rates, although relapses are common. The addition of etoposide has been suggested to improve outcomes, and incorporating the CD30-targeting immunoconjugate brentuximab vedotin into cyclophosphamide, doxorubicin, prednisone (CHP) chemotherapy regimens has resulted in improved progression-free survival (PFS) compared to standard CHOP (cyclophosphamide, doxorubicin, vincristine, prednisone) therapy [23]. In contrast, EBV+ NT/NKCL is considered to be an aggressive lymphoma. Currently, there is no standard treatment for EBV+ NT/NKCL. The clinical outcomes of patients treated with the CHOP regimen are poor [3,12,24]. Recent reports indicate that L-asparaginase-based regimens, such as SMILE (steroid, methotrexate, ifosfamide, L-asparaginase, and etoposide) and P-Gemox are effective against advanced-stage ENKTL [25]. Although there are no specific reports on the use of the SMILE regimen for nodal EBV+ NT/NKCL, and further research is necessary, it is speculated to be superior to CHOP. Unfortunately, our case 1 did not show a good response to P-Gemox for the relapsed EBV+ NT/NKCL. Additionally, upregulation of PD-L1 mRNA has been observed in EBV+ NT/NKCL, and anti-PD-1 immunotherapy has shown effectiveness in ENKTL patients [26,27], highlighting the potential therapeutic implications of anti-PD-1 treatment in nodal EBV+ NT/NKCL. It is also worth investigating whether including brentuximab in the treatment of CD30-positive EBV+ NT/NKCL will improve clinical outcomes. Further clinical studies focusing on the management of EBV+ NT/NKCL and ALK-negative ALCL based on their genetic profiles and other unique pathological characteristics may provide critical insights to improve the clinical outcomes of these patients.

## 4. Conclusions

EBV+ NT/NKCL may show morphologic and immunophenotypic features that closely mimic ALK-negative ALCL, including strong and uniform CD30 expression. Because EBV+ NT/NKCL is an aggressive disease with significant differences in the clinical management compared to ALK-negative ALCL, it is critical to identify cases of EBV+ NT/NKCL by including EBV tests (i.e., *EBER* staining of histologic sections and EBV serologic studies) in the diagnostic workup of cases in which a diagnosis of ALK-negative ALCL is being considered.

## Data Availability

Data are unavailable due to privacy.

## Supplementary Materials

The following supporting information can be downloaded at: https://www.mdpi.com/article/10.3390/hematolrep16020031/s1, Materials and Methods.
