# Supplementary material for: EBV-Positive Nodal T- and NK-Cell Lymphoma Mimicking Anaplastic Large Cell Lymphoma: A Case Report
Source: Hematol Rep. 2024 May 23;16(2):308–16. doi: 10.3390/hematolrep16020031 (PMC11203248; doi:10.3390/hematolrep16020031)
Supplement: Supplementary file 1 [file hematolrep-16-00031-s001.zip › hematolrep-2938971-supplementary.pdf]

Materials and Methods

Diagnosis and classification

The diagnosis and classification of T-cell lymphomas are based on the 5th edition World Health Organization Classification of Tumors of Hematopoietic and Lymphoid Tissues (WHO-HAEM5) [1] and the 2022 International Consensus Classification (ICC) [2].

All the laboratory tests described in the case study were established and validated as clinical diagnostic assays with performance characteristics meeting the United States Clinical Laboratory Improvement Amendments (CLIA) standard.

Immunohistochemical stains

Antibodies

Antigen	Antibody clone	Manufacturer/producer
ALK1	D5F3	Cell Signaling
CD2	11F11	Leica
CD3	LN10	Leica
CD4	4B12	Leica
CD5	4C7	Leica
CD7	LP15	Leica
CD8	C8/144B	Dako
CD15	Carb-3	Dako
CD25	4C9	Leica
CD30	Ber-H2	Dako
CD56	CD564	Leica
GATA-3	L50-823	BIOCARE
Ki-67	MIB-1	Dako
PD1	NAT105	Abcam
Perforin	5B10	Leica
TCRβ (βF1)	8A3	Life Technologies
TCRγ	GAMMA 3.20	Life Technologies
TIA-1	2G9A10F5	Beckman Coulter

Immunohistochemical stains were performed on Leica Bond III Automated IHC and ISH Staining System (Leica Biosystems, Deer Park, IL, USA) according the manufacturer's instructions using Bond™ Polymer Refine Detection Kit (Leica cat# DS9800) and antibodies at dilution pre-titrated during clinical test validation.

Flow cytometry

CD2, CD3, CD4, CD5, CD7, CD8, CD15, CD26, CD45, CD56, TCRαβ and TCRγδ antibodies were purchased from Becton Dickinson (Franklin Lakes, NJ, USA). The antibody-conjugated fluorochromes utilized were: CD2-V450, CD3-PE-Cy7, CD4-PerCP-Cy5.5, CD5-FITC, CD7-BV605, CD8-APC-H7, CD15-V450, CD26-PE, CD45-V500C, CD56-APC-R700, TCRαβ-APC and TCRγδ-PE.

Multi-color flow cytometry immunophenotyping (MFC) was performed on tissue samples using Becton Dickinson (BD) FACSCanto II flow cytometer. The tissue sample was bulk-lyzed and stained to single-cell suspension, data was acquired using BD FACSDiva software (50,000 events were acquired) and analyzed using Kaluza C 1.1 software (Beckman Coulter, USA).

In situ hybridization of Epstein-Barr virus-encoded small RNAs (*EBER*)

EBER staining was performed on Leica Bond III Automated IHC and ISH Staining System (Leica Biosystems, Deer Park, IL, USA) according the manufacturer's instructions using reagents provided by Leica, including Bond™ Enzyme Pretreatment Kit (Leica cat# AR9551), *EBER* Probe (Leica cat# ISH5687-A), RNA Negative Control Probe (Leica cat# ISH5950-A), RNA Positive Control Probe (Leica cat# ISH5894-A), and Bond™ Wash Solution (Leica cat# AR9590).

Quantitative polymerase chain reaction (PCR) detection of EBV DNA

The EBV DNA quantitative test is established with Qiagen (Germantown, MD 20874, USA) analyte specific reagents (ASR), including EBV Primer ASR (cat #1083003), EBV Probe ASR (cat #1083005), PCR Solution EL MBA (cat #1083007), and EBV Positive Control (cat #1083010). Briefly, DNA from whole blood was extracted from patient sample using MagNA Pure 96 DNA and Viral NA Small Volume Kit (cat# 06543588001, Roche Diagnostics, Indianapolis, IN, USA). Real-time PCR was performed on the ABI 7500 (ThermoFisher, Waltham, MA, USA). The International Units (IU) were converted from cycle threshold (Ct) of patient sample and preestablished calculation based on the standard curve.

Fluorescent *in situ* hybridization (FISH) for *ALK*, *P63* and *DUSP22*

The FISH tests were performed at Mayo Clinic Laboratories: https://www.mayocliniclabs.com/test-catalog/overview/65911. Accessed 24 April 2024.
